# Supplementary material for: The Influence of Community Feeders and Commercial Food Outlets on the Spatial Distribution of Free-Roaming Dogs—A Photographic Capture and Recapture Study
Source: Animals (Basel). 2023 Feb 24;13(5):824. doi: 10.3390/ani13050824 (PMC10000244; doi:10.3390/ani13050824)
Supplement: Supplementary file 1 [file animals-13-00824-s001.zip › Material Supplementar - Figures S1 and S2 tabela removida.pdf]

Figure S1 – Clusters of free-roaming dogs and sites of community feeders and commercial food outlets during in each of the five sampling efforts out in an urban area located in Divinópolis, Minas Gerais, Brazil

Figure S2 – Clusters of free-roaming dogs and sites of community feeders and commercial food outlets stratified by sex in each of the five sampling efforts out in an urban area located in Divinópolis, Minas Gerais, Brazil.

Figure S1 – Clusters of free-roaming dogs and sites of community feeders and commercial food outlets during in each of the five sampling efforts out in an urban area located in Divinópolis, Minas Gerais, Brazil.

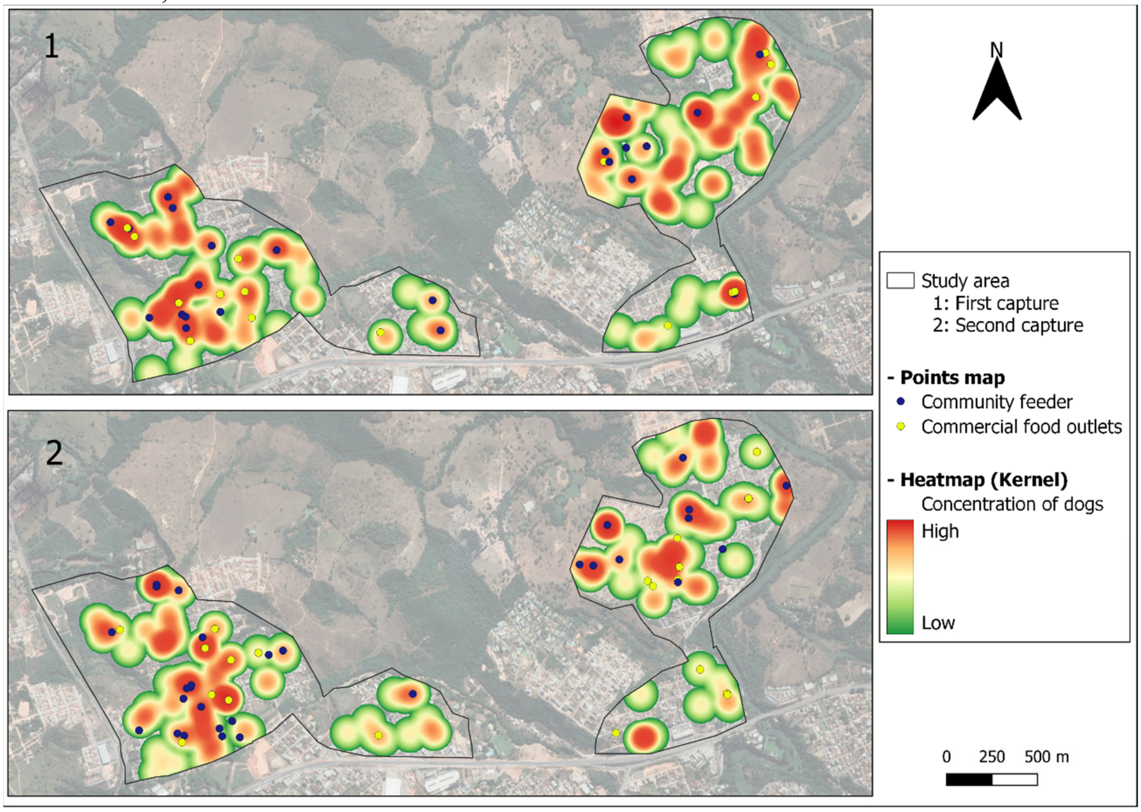

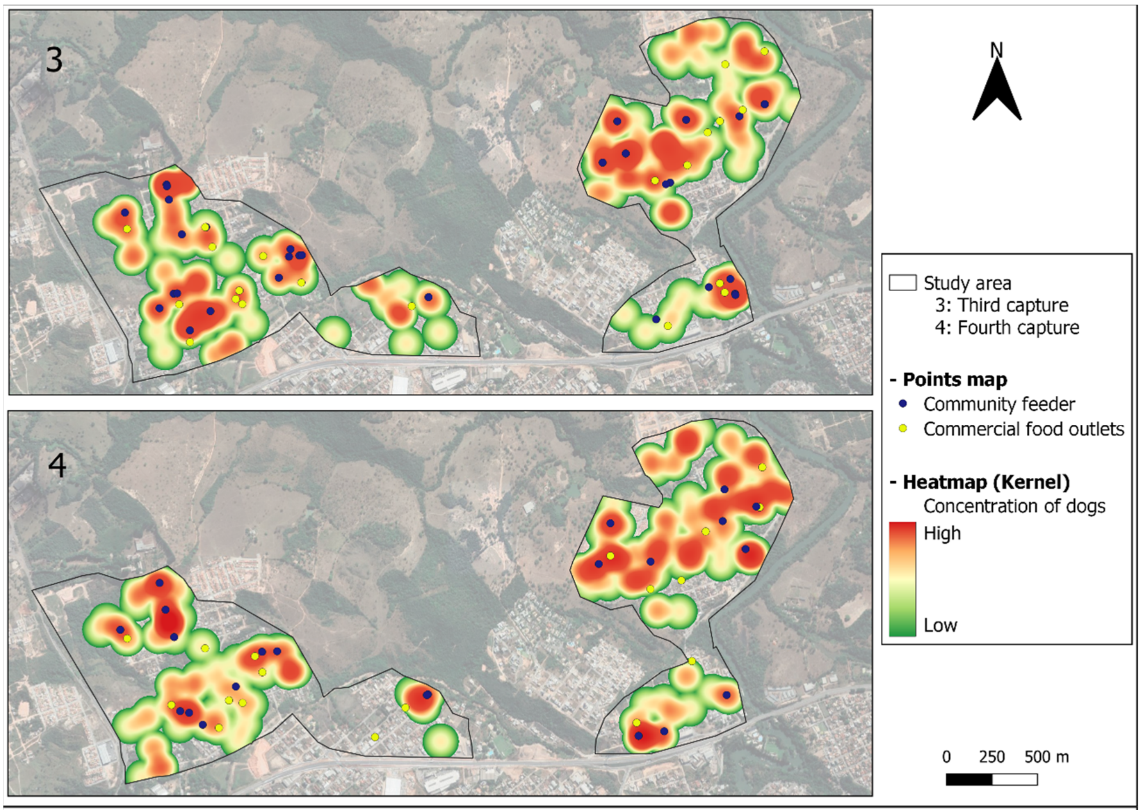
3
4
N
Study area
3: Third capture
4: Fourth capture
- Points map
Community feeder
Commercial food outlets
- Heatmap (Kernel)
Concentration of dogs
High
Low
0 250 500 m

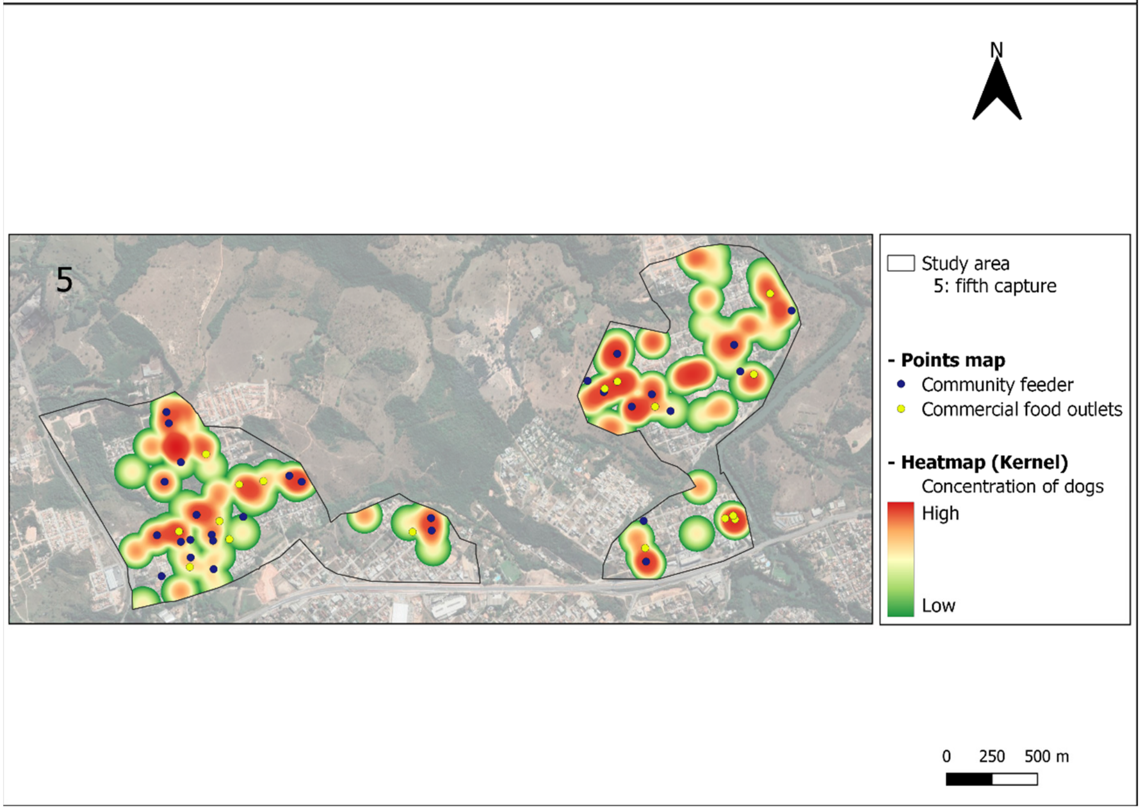
N
5
Study area
5: fifth capture
- Points map
Community feeder
Commercial food outlets
- Heatmap (Kernel)
Concentration of dogs
High
Low
0 250 500 m

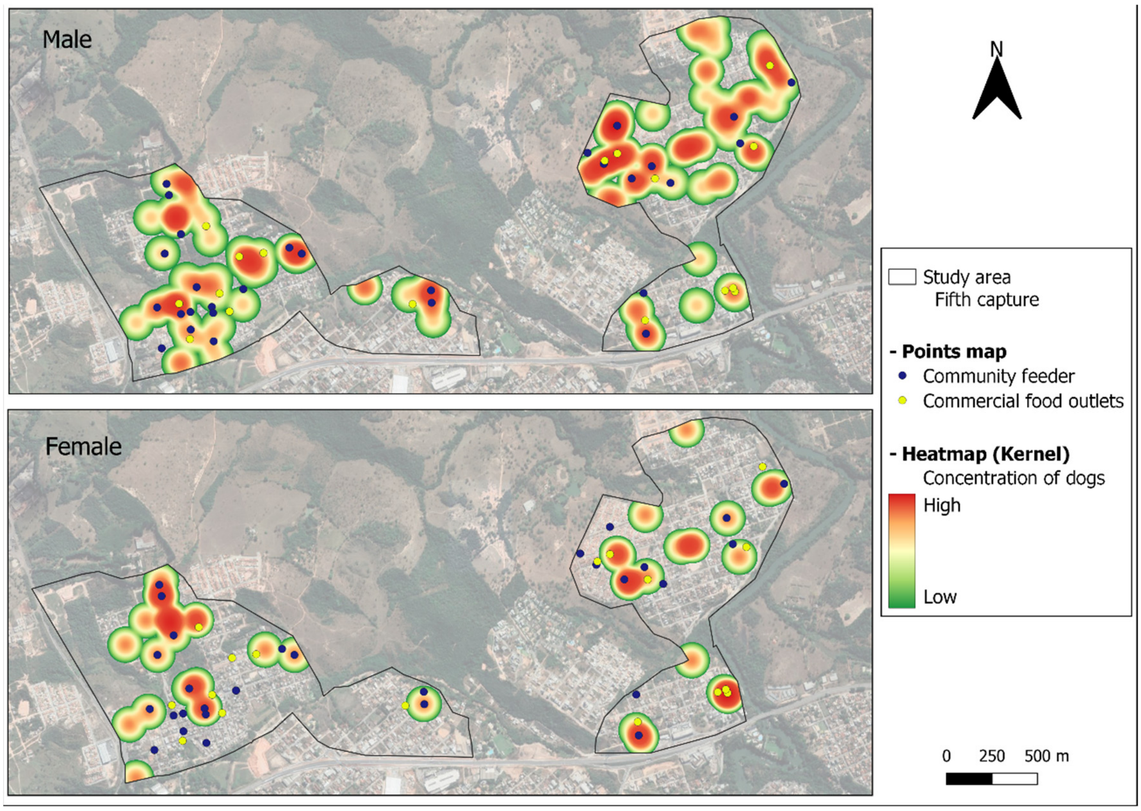

Male
Female
N
Study area
Fifth capture
- Points map
Community feeder
Commercial food outlets
- Heatmap (Kernel)
Concentration of dogs
High
Low
0 250 500 m

Figure S2 – Clusters of free-roaming dogs and sites of community feeders and commercial food outlets stratified by sex in each of the five sampling efforts out in an urban area located in Divinópolis, Minas Gerais, Brazil.

1

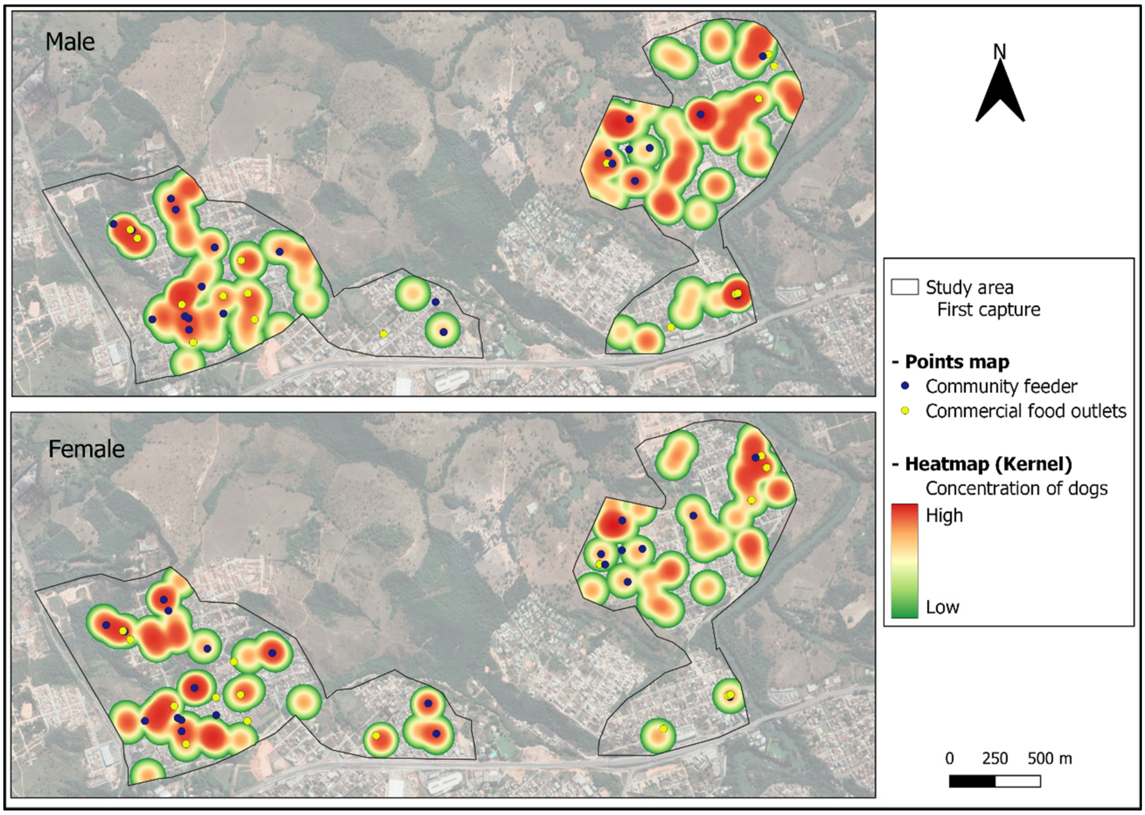

2

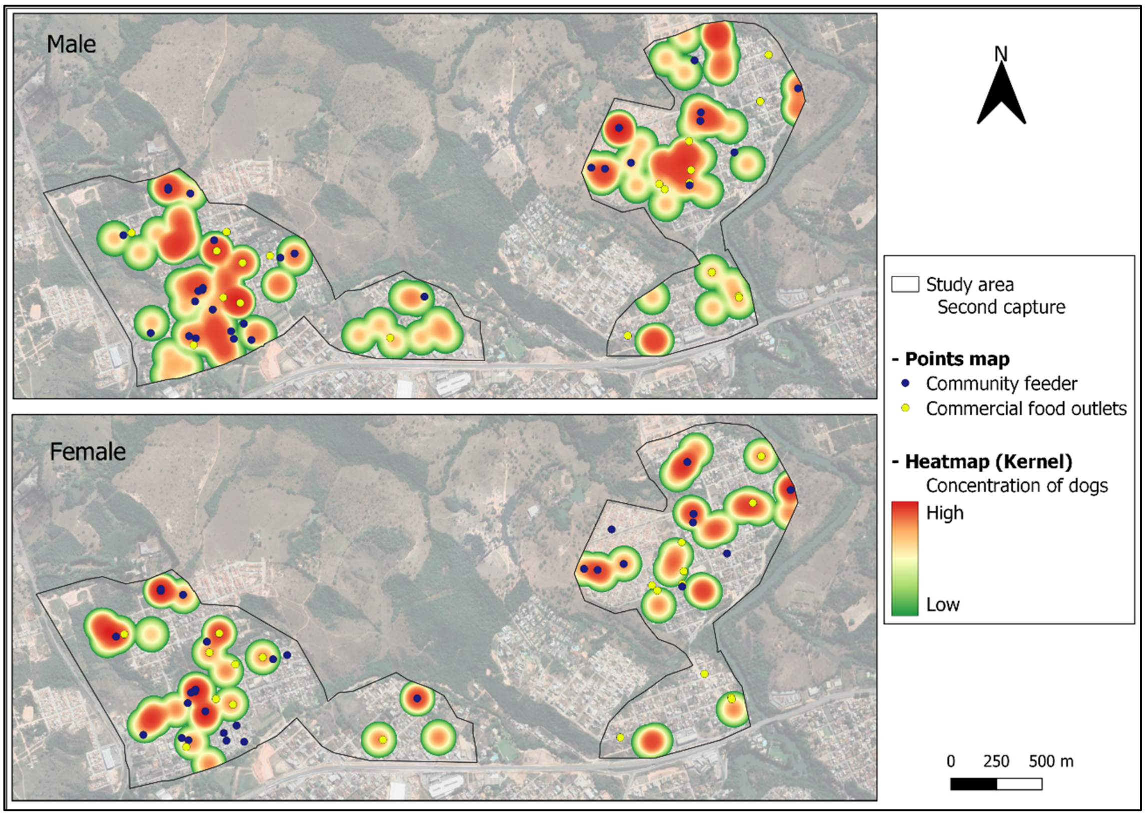
Male
Female
N
Study area
Second capture
- Points map
Community feeder
Commercial food outlets
- Heatmap (Kernel)
Concentration of dogs
High
Low
0 250 500 m

3

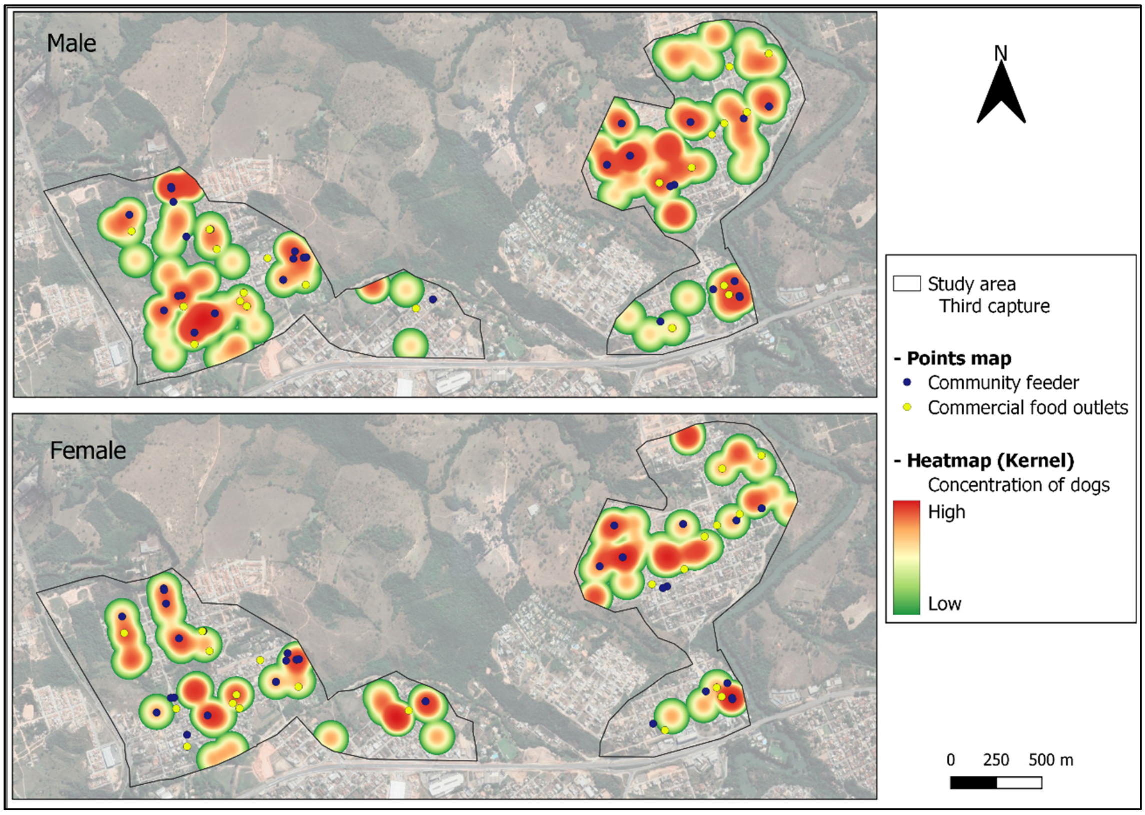
Male
Female
N
Study area
Third capture
- Points map
Community feeder
Commercial food outlets
- Heatmap (Kernel)
Concentration of dogs
High
Low
0 250 500 m

4

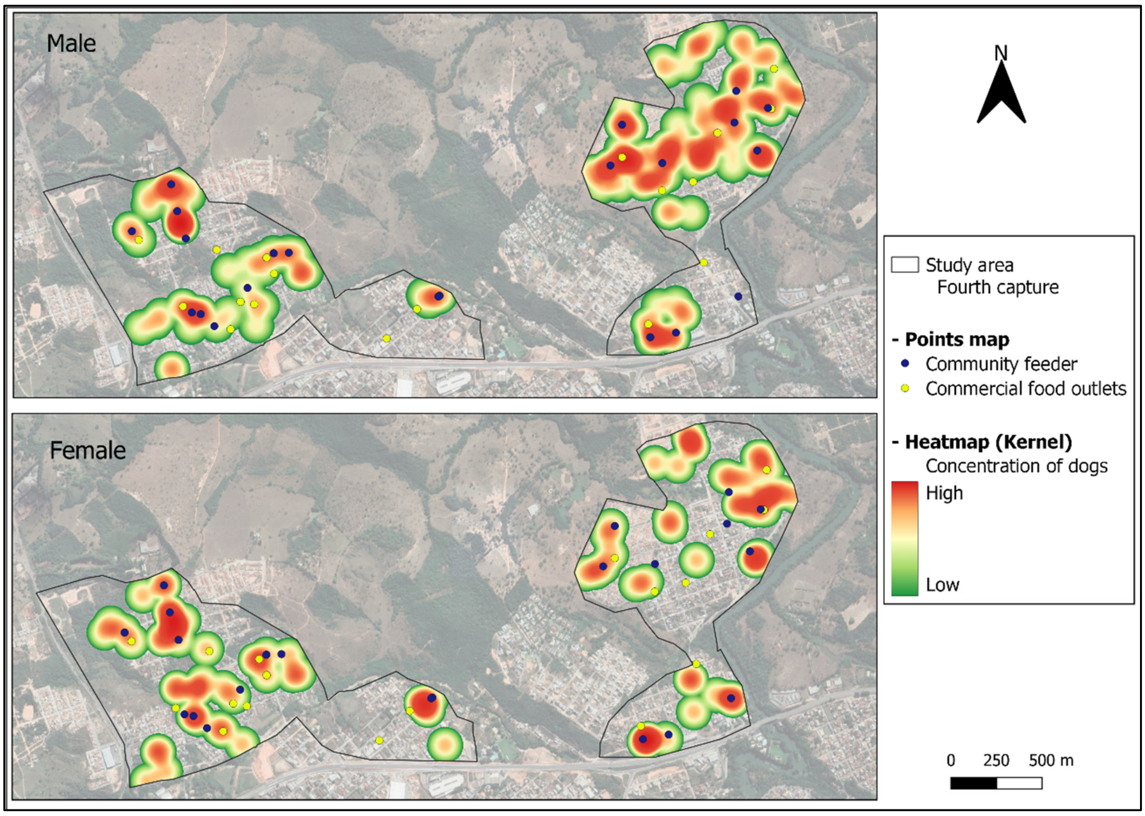
Male
Female
N
Study area
Fourth capture
- Points map
Community feeder
Commercial food outlets
- Heatmap (Kernel)
Concentration of dogs
High
Low
0 250 500 m

5

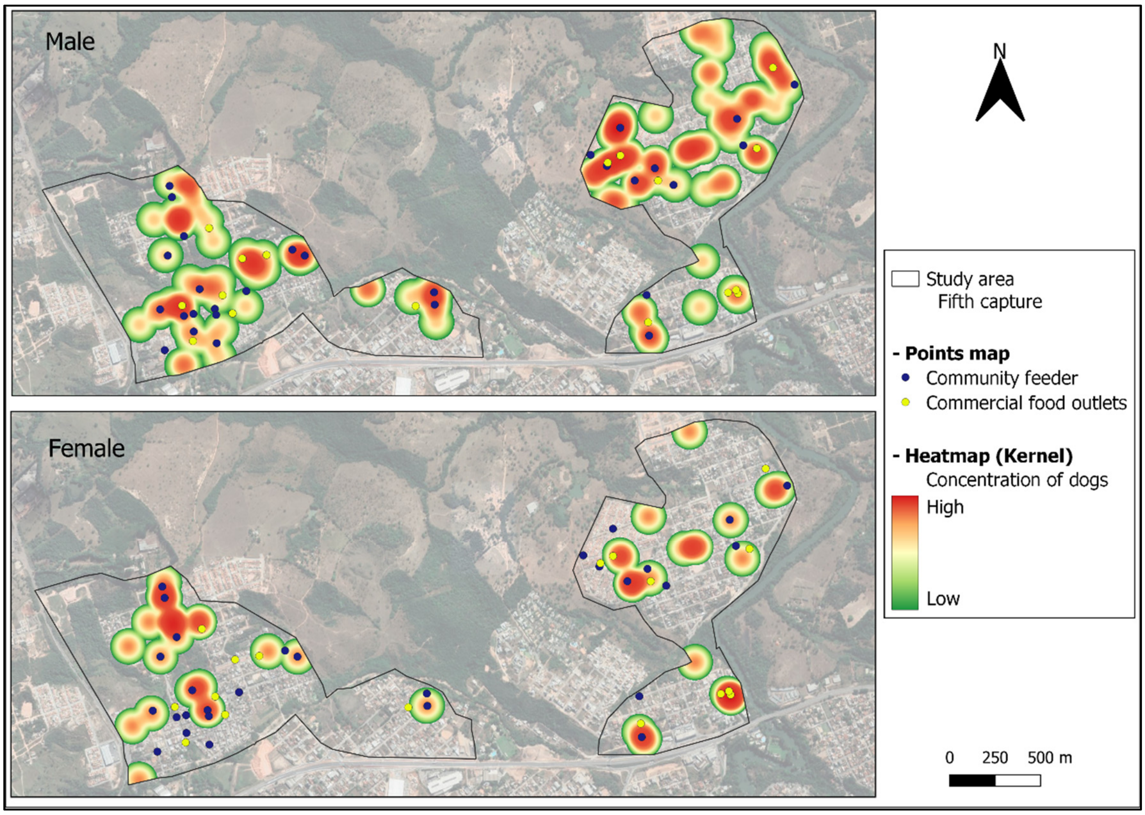
Male
Female
N
Study area
Fifth capture
- Points map
Community feeder
Commercial food outlets
- Heatmap (Kernel)
Concentration of dogs
High
Low
0 250 500 m
